# Aromaticity: what does it mean?

**DOI:** 10.1007/s40828-015-0012-2

**Published:** 2015-06-10

**Authors:** T. M. Krygowski, H. Szatylowicz

**Affiliations:** 1 Department of Chemistry, Warsaw University, Pasteura 1, 02-093 Warsaw, Poland; 2 Faculty of Chemistry, Warsaw University of Technology, Noakowskiego 3, 00-664 Warsaw, Poland

**Keywords:** Aromaticity, HOMA, NICS, Resonance energy, Pi-electron delocalization, Ring current

## Abstract

Aromaticity/aromatic belongs to one of the most useful and popular terms in organic chemistry and related fields. However, aromaticity is not an unambiguous term; therefore, its definition is enumerative. The criteria are based on energy (increased stability), molecular geometry (very low bond lengths alternation), magnetism (induction of the diatropic ring current by external magnetic field) and reactivity (tendency to maintain π-electron structure in chemical reactions). The energetic criterion is based on resonance energy and aromatic stabilization energy, whereas harmonic oscillator model of aromaticity—on molecular geometry. Magnetism-based criteria are illustrated by local indicators (for individual rings): nucleus independent chemical shifts and proton nuclear magnetic resonance chemical shifts as well as the global aromaticity index—exaltation of the magnetic susceptibility. For selected homo- and hetero-cyclic compounds, illustrative data are presented in tables, which allow the comparison of the above-mentioned indices. Finally, examples of agreements or disagreements between these various aromaticity indices are presented for few representative cases.

**Electronic supplementary material:**

The online version of this article (doi:10.1007/s40828-015-0012-2) contains supplementary material, which is available to authorized users.

## Introduction

**Fig. 1:**
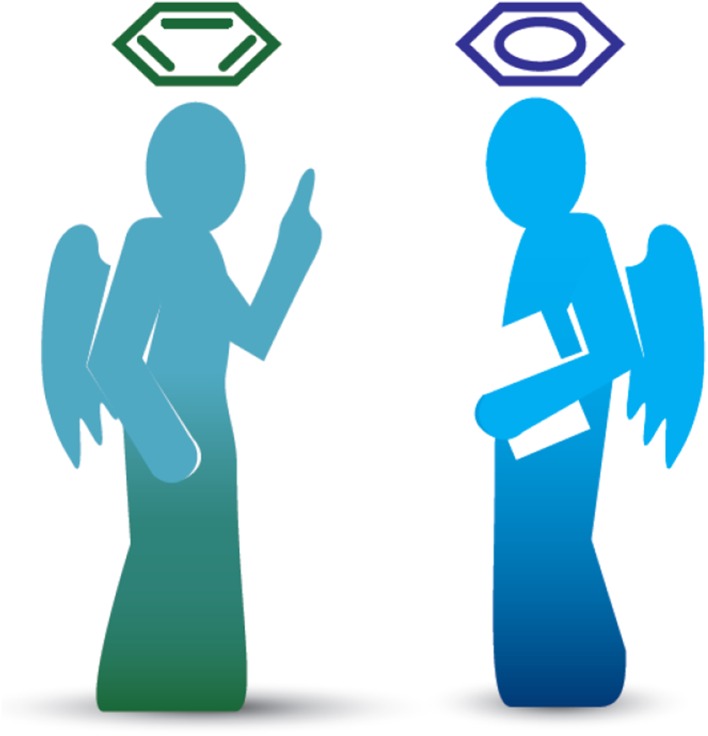
Aromaticity as a problem of π-electron delocalization

Aromaticity is a very frequently used term in chemistry and in related fields. Statistically, every day ~30 papers appear in which the terms ‘aromatic/aromaticity’ are used in either their titles or abstracts or the keywords [1]. Numerous organic compounds are either aromatic or contain aromatic fragments. The definition of aromaticity is enumerative in nature, *i.e*. it is described by a collection of physicochemical properties determining specific features of cyclic or polycyclic π-electron molecules [2–4]. The following features are accepted as decisive for defining a molecule to be aromatic (Fig. 1):

**Scheme 1:**
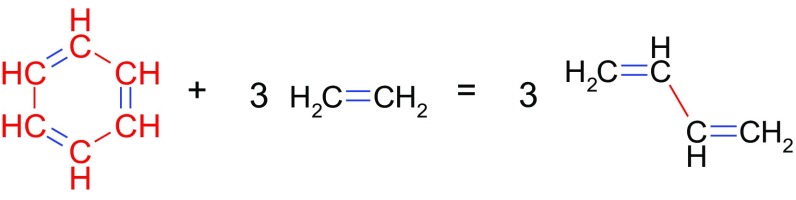
Homodesmotic reaction

1. as already mentioned it has to be cyclic or polycyclic;
2. its bond lengths exhibit very low bond lengths alternation;
3. it should be more stable than its acyclic analogue;
4. in external magnetic field it shows an increased diamagnetic susceptibility and diatropic (low field) chemical shifts of exocyclic protons in ^1^H NMR spectra, due to magnetic field induced ring current;
5. it more easily undergoes substitution reactions than the addition ones.

These criteria of aromaticity presented above may have some numerical representation, very often termed “aromaticity indices”.

## Energetic measure of aromaticity

Already in the nineteenth century, it was known that aromatic compounds (mainly benzene) are much more resistant to chemical reactions than their acyclic analogues [5]. First quantitative description of aromaticity was proposed in 1933 by introduction of a thermodynamic term, namely the resonance energy, RE [6] *i.e.* the energy by which the aromatic compound is more stable than its virtual olefinic analogue. In the case of benzene, this analogue is a virtual compound with three single and three double bonds. Estimated RE for benzene amounts to 36 kcal/mol. A very similar value was experimentally determined by Kistiakowsky et al. [7] through calorimetric measurements of heats of hydrogenation of benzene and cyclohexene [8].

Later, the term RE was replaced by more precisely defined aromatic stabilization energy (ASE) which is estimated by the use of either isodesmic [9] or more precise homodesmotic [10, 11] reactions. The latter is defined as a virtual reaction leading to products with the same number of CH bonds and the same numbers of atoms in the appropriate hybridization states (see Scheme 1; Table 1 in which representative cases of homodesmotic reactions are presented).

**Table 1:**
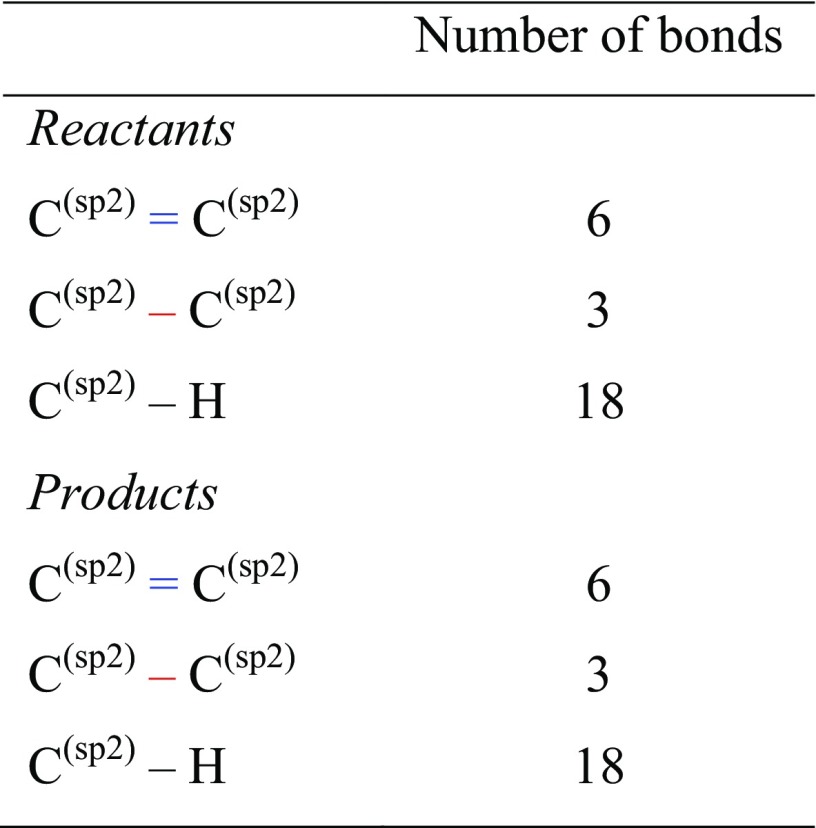
Types of bonds for homodesmotic reaction shown in Scheme 1

C_6_H_6_ + 3 CH_2_CH_2_ = 3 *trans* CH_2_CHCHCH_2_

C_6_H_6_ + 3 CH_2_CH_2_ = 3 *cis* CH_2_CHCHCH_2_

ASE = 23.2 kcal/mol

ASE = 33.6 kcal/mol

Here the problem of an appropriate reference for the stability of aromatic molecules arises. Table 2, data taken from [12], shows that ASE values depend dramatically on the choice of the computation method of computation and of the selection of the reference system [12].

**Table 2:**
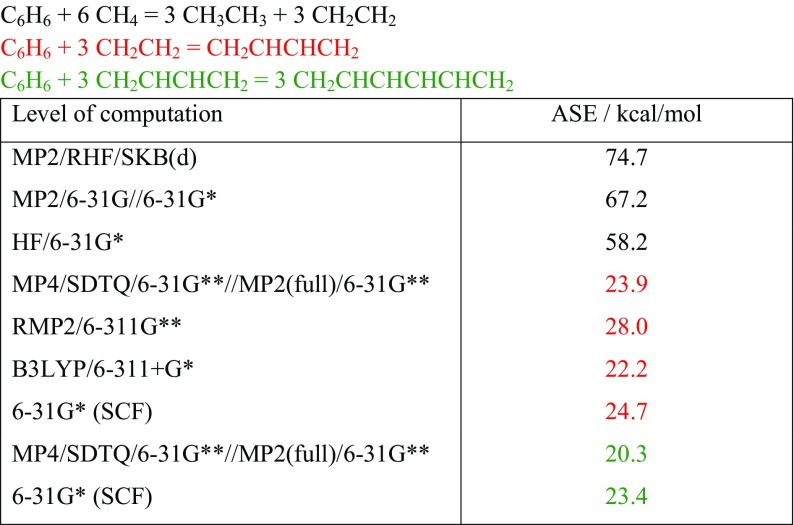
Stabilization energies^a^ of ISODESMIC and  reactions:

^a^Data taken from [12]

Apart from these limitations, the ASE approaches are mostly applied to π-electron hydrocarbons. They are much less effective in the case of heterocyclic systems where the problem of the reference system is much more complicated.

For tautomers and isomers yielding reliable information on energetic relations between different chemical species, a direct comparison of energy is possible, without the necessity of applying any kind of ASE procedure.

Undoubtedly the energetic criterion is very important but it describes only stability of the whole molecule. However, it is well known that in polycyclic π-electron molecules, rings of different stability can co-exist. Phenanthrene is a good example here, since its central ring is more reactive. Addition to the C9C10 bond of the central ring is relatively easy, whereas all positions in the two other rings are chemically more inert. Several procedures were proposed for determining energetic characteristics of individual rings, which are based on their geometry [13–15], for review see [16].

### HOMA: geometry-based aromaticity index

The next criterion of aromaticity is directly based on the molecule geometry. In strongly aromatic compounds the bond lengths either do not alternate or their alternation is very weak. Quantitatively it was first considered by Julg and Francois [17] who defined a numerical characteristic of aromaticity as a function of variance of the perimeter bond lengths in a molecule. Unfortunately application of this approach was limited to hydrocarbons only, since there is no possibility to estimate an averaged bond length for heterocyclic molecules. Hence an improvement was necessary to make the concept more general. It was done by replacing the averaged bond length *R*_av_ by a hypothetical optimal bond length *R*_opt_ proper for fully aromatic molecules [18, 19]. This geometry based aromaticity index has the form expressed by equation:

1 $$ {\text{HOMA}} = 1 - \frac{{\alpha_{j} }}{n}\sum\limits_{i}^{n} {(R_{{{\text{opt,}}j}} - R_{j,i} )^{2} } . $$

where α_*j*_ is a parameter (normalization constant) dependent on the type of a given bond (*j* stands for CC, CN, CO, CP, CS, NN, NO, etc.) and is estimated empirically from the lengths of their optimal (*R*_opt_), single (*R*_s_) and double (*R*_d_) bonds. The *R*_opt_ denotes the length of the bond for which extension to the single bond and compression to the double bond costs energetically the same. Energy of compression or extension is estimated by the use of a harmonic oscillator approach. Table 3 presents all data necessary for the HOMA model application [16] to molecules with heteroatoms involving bonds.

**Table 3:** Reference bond lengths *R*_s_ and *R*_d_, and appropriate *R*_opt_ and α values used in the HOMA index

Type of bond	*R*_s_/Å	*R*_d_/Å	*R*_opt_/Å	α	References
BB^a^	1.6474	1.5260	1.5665	244.147	[20]
BB^w a^	1.6474	1.5260	1.5693	250.544	[20]
BC^exp b^	1.5472	1.3616	1.4235	104.507	[21]
BC^theo b^	1.5542	1.3796	1.4378	118.009	[21]
BC^theo/w b^	1.5542	1.3766	1.4386	118.618	[21]
BN^c^	1.564	1.363	1.402	72.03	[22]
CC^d^	1.467	1.349	1.388	257.7	[23]
CN^e^	1.465	1.269	1.334	93.52	[23]
CO^f^	1.367	1.217	1.265	157.38	[23]
CP^g^	1.814	1.640	1.698	118.91	[23]
CS^h^	1.807	1.611	1.677	94.09	[23]
CSe^i^	1.959	1.7591	1.8217	84.9144	[24]
NN^j^	1.420	1.254	1.309	130.33	[23]
NO^k^	1.415	1.164	1.248	57.21	[23]

Reference systems used: ^a^ H_2_B-BH_2_ and HB=BH; ^b^ H_3_C-BH_2_ and H_2_C=BH; ^c^ H_3_B-NH_3_ and (*iso*Pr)_2_N=B=C(SiMe_3_)_2_, H_3_B-NH_3_ and H_2_B=NH_2_; ^d^ buta-1,3-diene; ^e^ H_2_N-CH_3_ and HN=CH_2_; ^f^ HCOOH monomer; ^g^ H_2_C=P-CH_3_; ^h^ S(CH_3_)_2_ and H_2_C=S, ^i^ H_3_C-SeH and H_2_C=Se; ^j^ (CH_3_)_2_C=N–N(CH_3_)_2_ and H_3_C–N=N–CH_3_;^ k^ CH_3_–O–N=O

It follows from the data of Table 1 that all π-electron systems with bonds presented there can be treated with the HOMA approach, provided that their reliable geometry is known.

Term (1) can be analytically transformed into a more detailed form [25] as (2), (3) and (4)

2 $$ {\text{HOMA}} = 1 - \frac{1}{n}\sum\limits_{i} {\alpha \left( {R_{\text{opt}} - R_{i} } \right)^{2} } = 1 - {\text{EN}} - {\text{GEO}} $$

where

3 $$ {\text{GEO}} = \frac{1}{n}\sum\limits_{i} {\alpha \left( {R_{\text{av}} - R_{i} } \right)^{2} } $$

and

4 $$ {\text{EN}} = \alpha \left( {R_{\text{opt}} - R_{\text{av}} } \right)^{2} $$

**Fig. 2:**
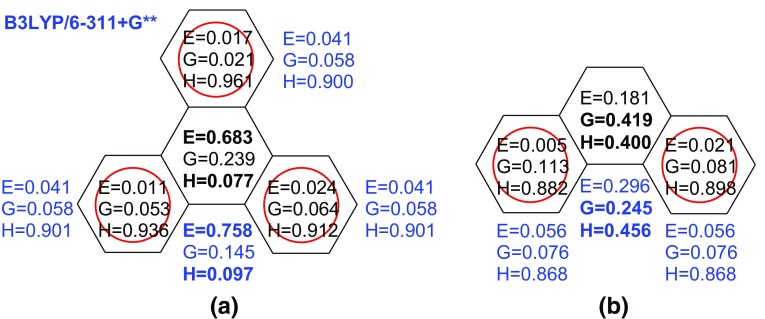
Dependence of the aromatic character of benzene ring on its topological environment in benzenoid hydrocarbons: **a** triphenylene and **b** phenanthrene; E, G and H denotes EN, GEO and HOMA parameters, respectively; values inside of the ring (taken from [26]) were obtained for experimental structures

Formulae (3) and (4) describe two structural factors deciding about aromaticity of a molecule in question. The GEO term describes the degree of bond length alternation—the greater GEO, the greater loss of the aromatic character due to an increase of alternation. This term is equivalent to Julg’s definition of aromaticity [17]. The second term, EN describes the loss of aromaticity due to lengthening of bonds over the mean length. According to Eq. (2) both terms lead to a decrease of the HOMA value. Figure 2 presents the results of the application of Eqs. (1), (2), (3) and (4) to phenanthrene and triphenylene [26].

Few interesting findings should be pointed out. First, the HOMA, EN and GEO terms based on experimental geometry are different for symmetrically equivalent rings. This is due to specific conditions of the molecular geometry determination by X-ray diffraction. If a molecule in a crystal lattice lies in a special position, *i.e.* coincides with a symmetry element, then its symmetrical property is maintained. However, if the position of the molecule and the symmetry element do not coincide, then its molecular environment in the crystal is no more symmetrical and hence different intermolecular interactions act on this molecule which would be symmetrical in the free state [27]. In Fig. 2 EN (E), GEO (G) and HOMA (H) values calculated from the experimental data are compared with those derived from purely computational (B3LYP/6-311 + G**) geometry. The observed differences seem to be significant in some cases, but the overall picture in both approaches is very similar.

In both molecules the central rings are significantly less aromatic than the peripheral ones. The HOMA values of the latter reach 0.9 and in the case of triphenylene even exceed this value. However, the decrease of HOMA in the phenanthrene central ring is predominantly associated with a greater value of the GEO than the EN term. This means that the dominant contribution to dearomatization comes from an increase of the bond length alternation. Definitely, this ring has a low aromatic character in line with its significant reactivity: addition to 9, 10 positions, for example. An opposite trend is observed for the central ring of triphenylene. Here the GEO term is much smaller than the EN term and the low HOMA value is due to lengthening of the central ring bonds. The central ring is then non-aromatic, and is known in Clar’s classification [28, 29] as the empty one, *i.e.* exhibiting a deficiency of pi electrons in the ring. Interestingly, when the geometry based estimation of energy of individual rings is applied [30], then the central rings in phenanthrene and triphenylene show bond energy (BE) values equal to 699.4 and 668.9 kcal/mol, respectively. All other rings in these molecules have the BE values between 715.6 and 725.2 kcal/mol. Definitely, the central rings have a lower energy content than the other ones, in line with the predictions resulting from their HOMA values. It is important to note that Eqs. (1)–(4) give an additional information of the reason of the observed aromaticity decrease. HOMA, EN and GEO values for selected homo- and heterocyclic compounds are presented in Table 4.

**Table 4:** HOMA EN and GEO values for selected homo- and heterocyclic compounds

	HOMA	EN	GEO	References
	0.86			[31]
	0.236			[32]
	0.75	0.04	0.21	[33]
	0.20	0.20	0.60	[33]
	0.72	0.03	0.25	[33]
	0.88			[31]
	0.998	−0.009	0.011	[34]
Benzene	0.979	0.021	0.000	[26]
Naphthalene	0.802	0.077	0.121	[26]
Cyclopentadiene	−0.778			[35]
	−0.381			[36]

### Magnetic-based aromaticity descriptors

**Fig. 3:**
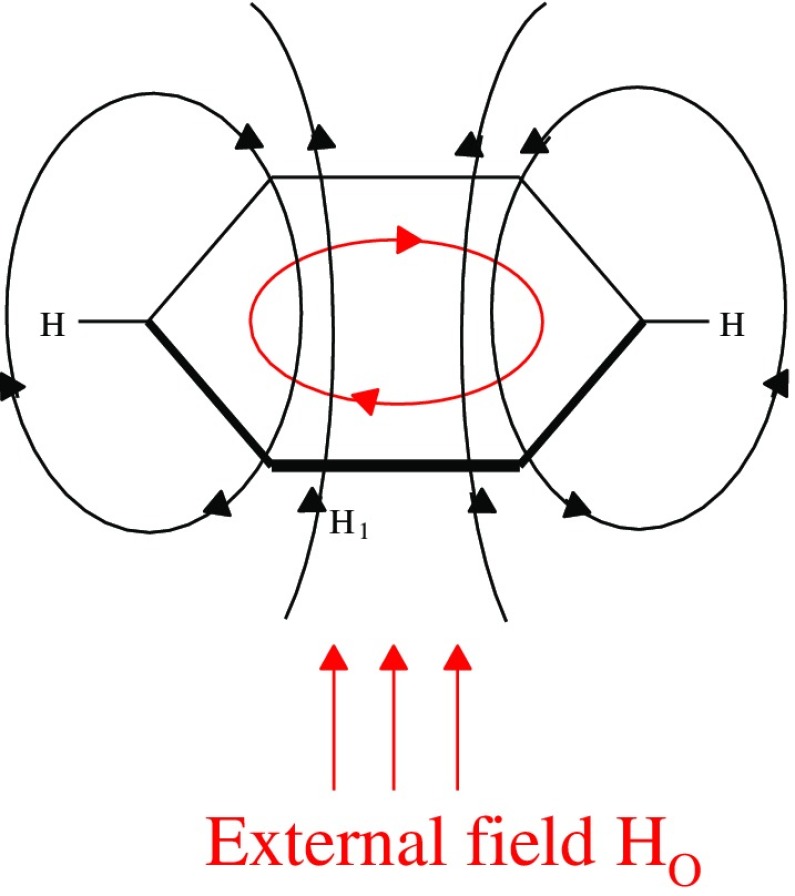
External magnetic field inducing an internal ring current, leading to characteristic “aromatic” ^1^H NMR shifts. Reprinted with permission from [12] Copyright 2005 American Chemical Society

Other criteria of aromaticity are based on specific magnetic properties of π-electron molecules. It is known from ^1^H NMR that chemical shifts for external protons in aromatic molecules are deshielded. Figure 3 illustrates it taking benzene as an example.

A term “aromatic chemical shifts” was even introduced for aromatic protons, which are larger (~7 ppm) than those measured for olephinic (~5 ppm) or for aliphatic (~1 ppm) protons [37]. ^1^H NMR chemical shifts may differ for various positions of protons as shown in the case of phenanthrene [38], see Table 5. This means that to some extent ^1^H NMR chemical shifts may serve as a local measure of aromaticity, having in mind that these data depend on the medium used for the measurements [38].

Another local measure of aromaticity was introduced by Schleyer et al. [29, 40]. These authors introduced a purely theoretical concept of nucleus independent chemical shift (NICS) that has later become one of the most popular characteristics of aromaticity. NICS is defined as a negative value of the absolute shielding measured in the center of a given ring [NICS(0), one angstrom above the center NICS(1)] or, alternatively, as the value of the perpendicular component of the tensor describing the shielding, NICS(1)_zz_. Table 6 presents NICS values for some π-electron ring systems; the more negative value of NICS, the more aromatic is the system [39]. As it can be easily noticed in some cases NICS values are in opposition to ASE or HOMA indices. For example, according to its NICS index, naphthalene is more aromatic than benzene. This problem arises from the fact, that NICS values depend on the size of a system being examined. Inspection of the data presented in Table 6 also leads to a conclusion that heterocyclic compounds such as pyrrole, thiophene and furan are inadequately described by this index since according to NICS values they are more aromatic than benzene—again against all other evidences (compare results Tables 4, 6).

**Table 5:** ^1^H NMR characteristics of phenanthrene; spectrum recorded in CDCl_3_ [38]

	Hydrogen	Chemical shift/ppm
	1	7.901
	2	7.606
	3	7.666
	4, 5	8.702
	9, 10	7.751

**Table 6:** Magnetic susceptibility exaltation (Λ) and NICS(0) values for selected homo- and heterocyclic compounds

	Λ/cgs ppm	Refs. for Λ	NICS(0)/ppm	Refs. for NICS(0)
Pyrrole	−6.5	[32]	−15.1	[39]
Phosphole	−1.7	[32]	−5.35	[40]
Thiophene	−7.0	[32]	−13.6	[39]
Furan	−2.9	[32]	−12.3	[39]
Benzene	−10.47	[41]	−9.7	[39]
Naphthalene	−20.98	[41]	−9.9	[39]
	−13.81	[41]	−7.6	[39]
Cyclopentadiene	−2.4	[39]	−3.2	[39]
Cyclohexane	−0.7	[39]	−2.2	[39]
Pentalene	34.59	[41]	18.1	[39]
	76.6	[39]	22.7	[39]
Cyclobutadiene	17.20	[41]	27.6	[39]

Apart from the above-mentioned local aromaticity characteristics, there are well known whole-molecule characteristics (named also as global aromaticity measures), accessible both experimentally and theoretically. The most important are: (1) anisotropy of magnetic susceptibility Δχ, [42] Eqs. (5), and (2) magnetic susceptibility exaltation Λ [43] Eq. (6).

5 $$ \Delta \chi = \chi_{cc} { - }\raise.5ex\hbox{$\scriptstyle 1$}\kern-.1em/ \kern-.15em\lower.25ex\hbox{$\scriptstyle 2$} \left( {\chi_{aa} + \chi_{bb} } \right) $$

and

6 $$ \Lambda = \chi_{M} {-}\chi_{{M{\prime }}} $$

where χ_*cc*_, χ_*aa*_ and χ_*bb*_ are the elements of the diagonalized magnetic susceptibility tensor and *c* is the out of plane direction for the planar molecule.

Both characteristics are relative in character. The former is a difference between the out of plane and the average in plane components (as reference) of the magnetic susceptibility tensor. The magnetic susceptibility exaltation is estimated in reference to the value for some non-aromatic (M′), artificial systems. The latter case resembles the resonance energy concept, where energy of a real system is related to some value for an artificial “olefinic analogue”.

Mills and Llagostera [41] found that the summation of aromatic and antiaromatic hydrocarbons values of NICS(1)_*zz*_ yields a very good correlation with the magnetic susceptibility exaltation.

### Effect of intra- and inter-molecular interactions on aromaticity of the ring

**Fig. 4:**
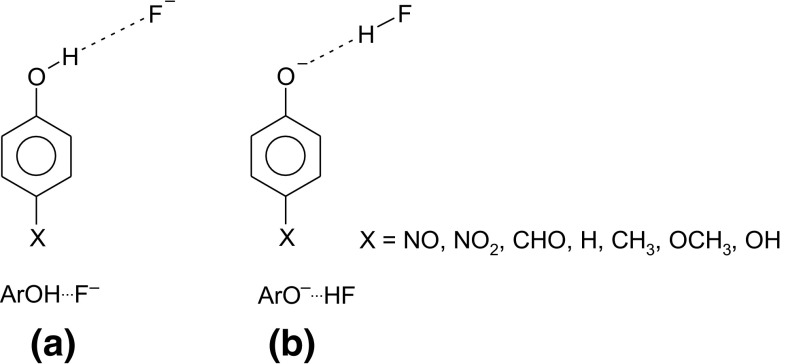
Structural scheme of the computational model

The dependence of benzene ring aromaticity on the substituent type and on the strength of intermolecular interactions in the case of phenol and phenolates is an interesting exemplification of the factors which can influence this property. It can be demonstrated by a computational model of approaching the hydroxyl group by F^−^ and the anionic (phenolate) form by HF [44] (see Fig. 4).

**Fig. 5:**
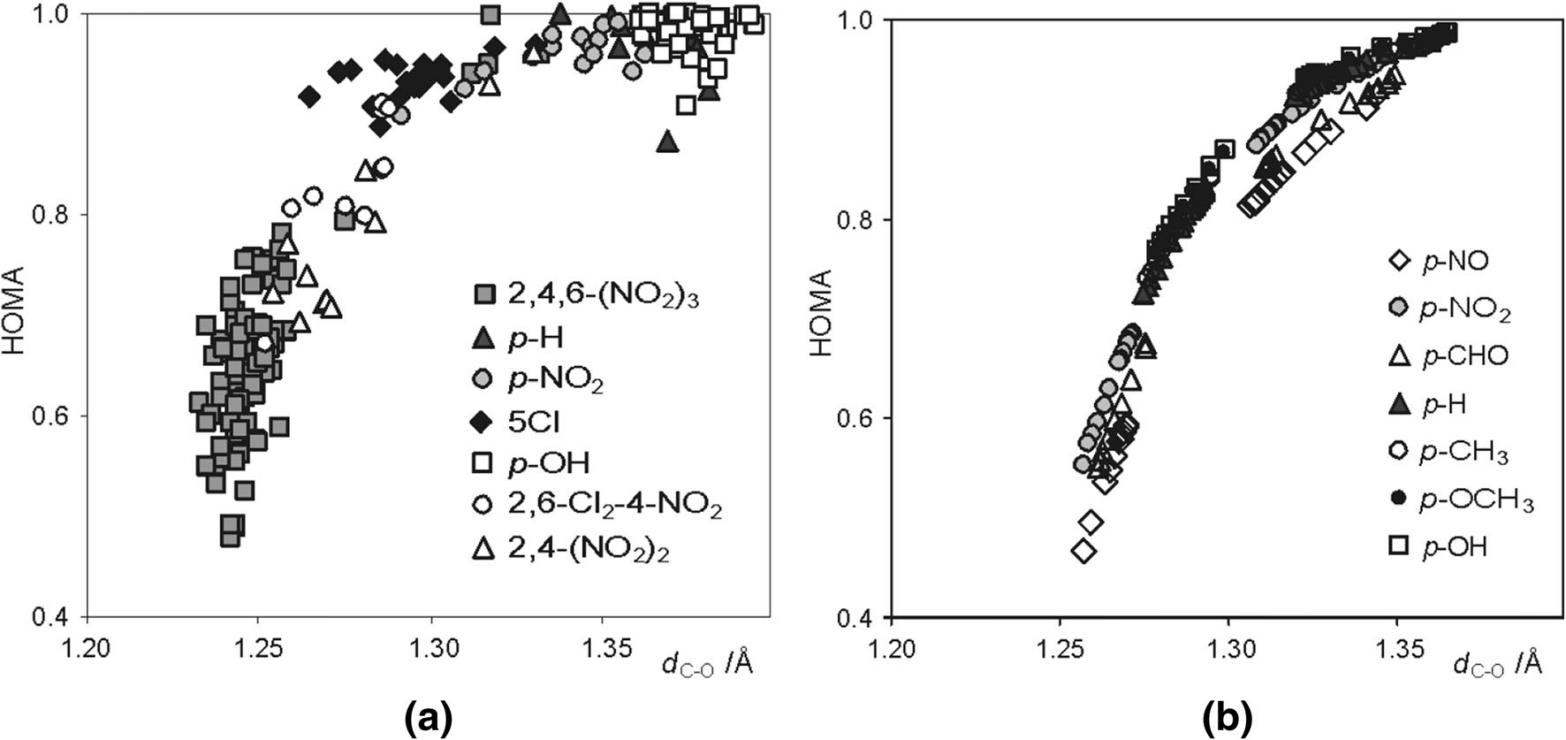
Dependence of aromaticity of phenyl ring (HOMA) on hydrogen bond strength, **a** X-ray data (CSD, 664 geometries) and **b** B3LYP/6-311 + G** results. Reprinted with permission from [45, 46]. Copyright 2004 and 2005 American Chemical Society

As it can be seen from the data presented in Fig. 5 the obtained HOMA indices for the benzene ring in substituted phenols and phenolates clearly correlate with the strength of the hydrogen bond, determined by the C–O bond length. Moreover, theoretically calculated and experimentally determined (data taken from Cambridge Structural Data Base) C–O bond lengths yield qualitative very similar pictures [45, 46]. Thus, strengthening of the H-bond in *p*-X-PhOH…F^−^ complexes (shortening of the C–O bond length) results in lowering of aromaticity, whereas for *p*-X-PhO^−^…HF systems the opposite trend is observed.

**Fig. 6:**
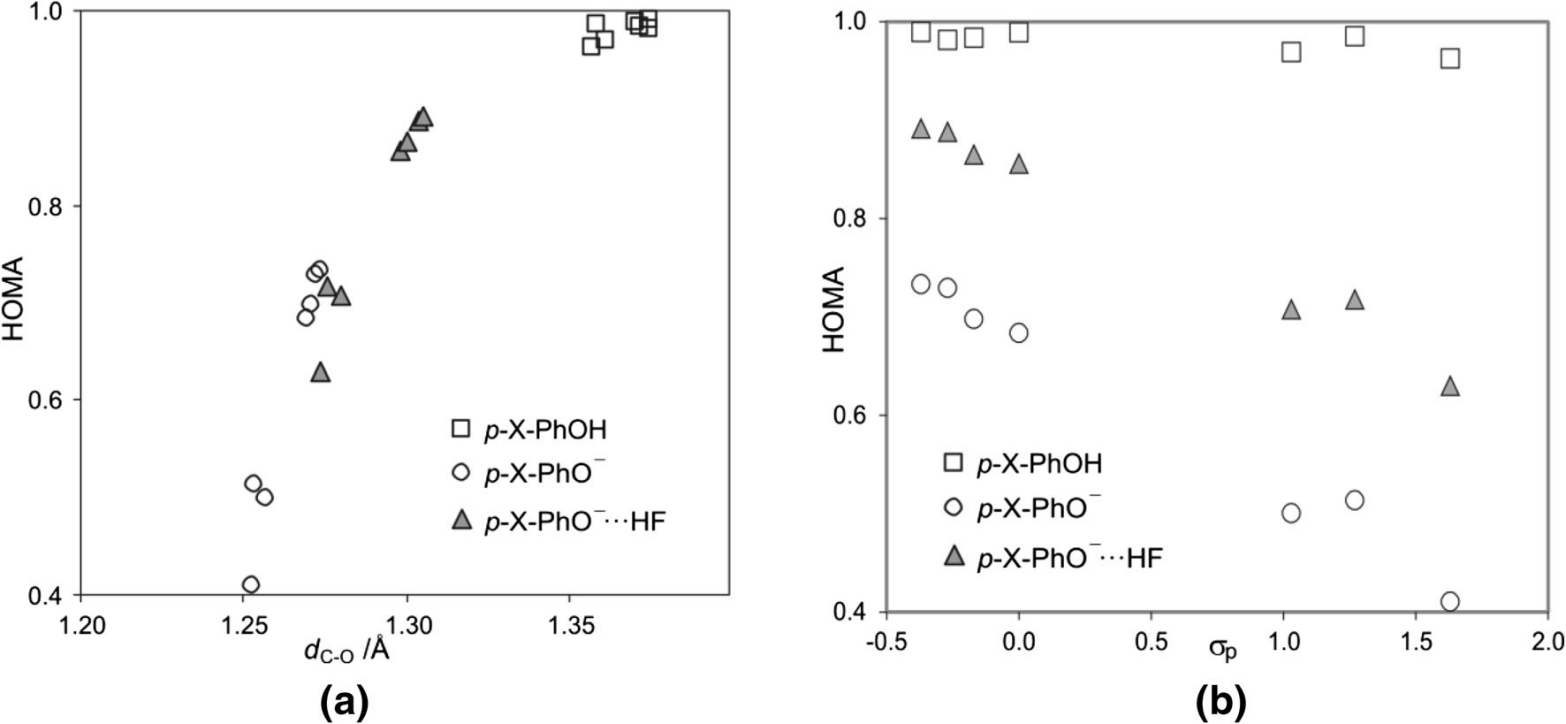
Dependence of aromaticity of phenyl ring (HOMA) on **a** the C–O bond length, *d*_C–O_, and **b** a substituent constant, σ_p_ (for electron accepting substituents σ_p_^−^ are used) for *para* substituted phenols, phenolates, and their H-bonded equilibrium complexes (*p*-X-PhO^−^…HF). Part **a** reprinted with permission from [46]. Copyright 2005 American Chemical Society

One of the most important problems in organic chemistry is impact of the substituents on the system in question. The classical approach to these problems is strongly related to substituted benzene derivatives and described by the fundamental theory introduced by Hammett [47], the most recent review is given in [48]. Application of the Hammett ideas for *para* substituted phenols, phenolates and their equilibrium H-bonded complexes is presented in Fig. 6.

**Fig. 7:**
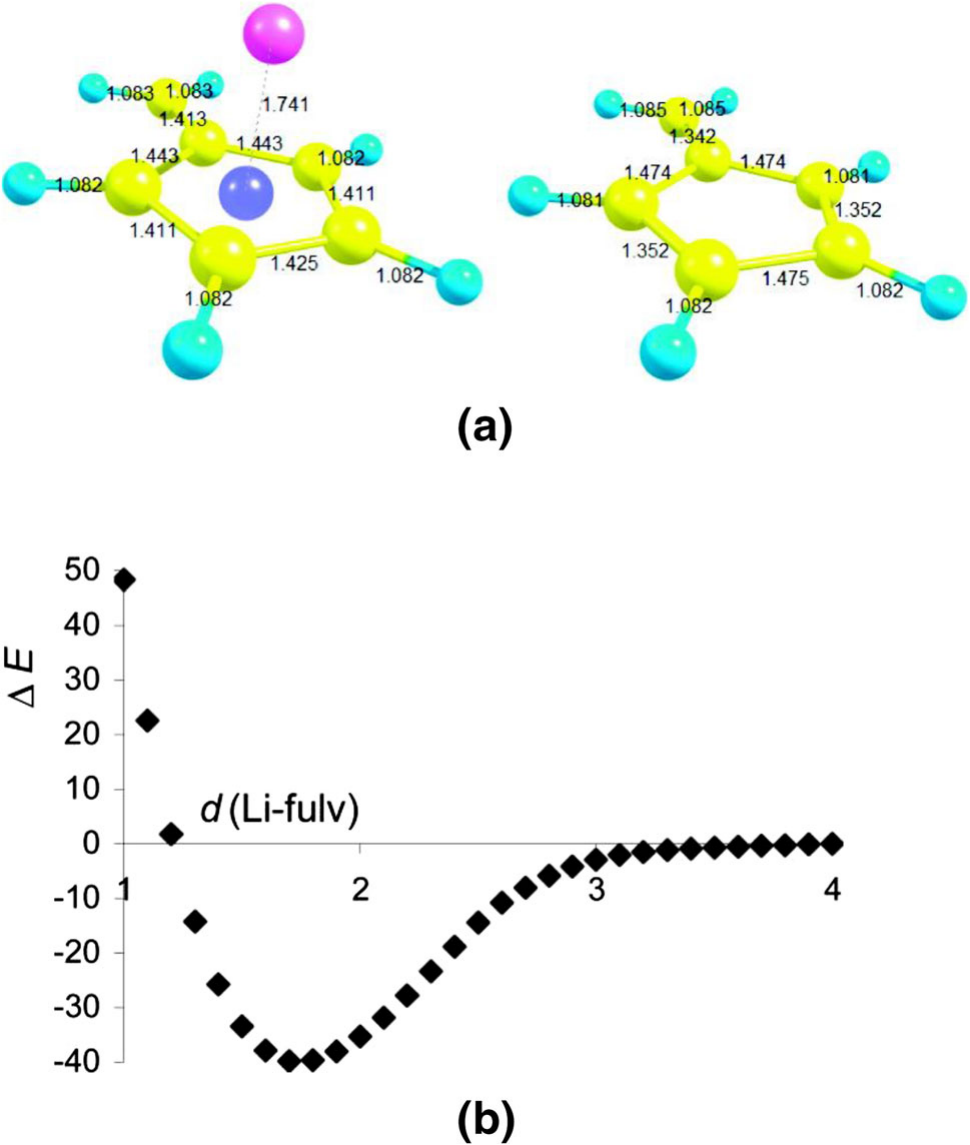
**a** Computed structures of the Li-fulvene complex and the free fulvene molecule [B3LYP/6-311++G(d,p) level]. **b** Relative energy, Δ*E* (kcal/mol), of the Li-fulvene complex relative to neutral fragments, as a function of the distance from Li to the ring center, *d*(Li-fulv) (Å). Reprinted with permission from [49]. Copyright 2010 American Chemical Society

It should be stressed that in some cases a qualitative agreement is encountered in the estimation of aromaticity by means of geometry based HOMA, energy and NICS’s indices. Interactions between fulvene lithium can be considered as an instructive example here [49]. Figure 7 presents energy the potential well obtained as a result of approaching the center of fulvene ring by Li. Fulvene is known as a nonalternant π-electron hydrocarbon [50, 51]. If the Li atom gets closer to its ring the stability of the resulting complex increases, up to ~40 kcal/mol in the equilibrium state. This is also manifested by the increase of aromaticity as evidenced by the change of HOMA from ~−0.3 to ~0.6 and NICS(0) from 0.94 to −11.15.

### Multidimensional character of aromaticity

**Fig. 8:**
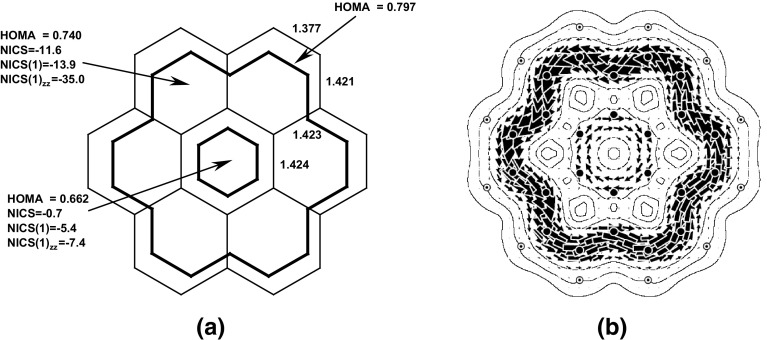
**a** Bond lengths and the aromaticity descriptors: HOMA, NICS, NICS(1) (calculated 1 Å above the molecular plane), and NICS(1)_*zz*_ (the component of NICS(1) corresponding to the principal axis perpendicular to the ring plane) for fragments of coronene. **b** Map of π-current density in coronene. Diatropic and paratropic circulations are shown anticlockwise and clockwise, respectively. Reprinted with permission from [52]. Copyright 2010 American Chemical Society

**Fig. 9:**
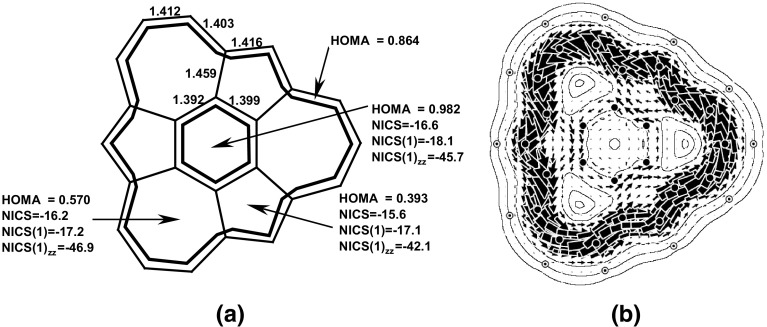
**a** Bond lengths and the aromaticity descriptors: HOMA, NICS, NICS(1), and NICS(1)_*zz*_ for isocoronene. **b** Map of π-current density in isocoronene. The NICS descriptors follow the notation used in Fig. 7. Reprinted with permission from [52]. Copyright 2006 American Chemical Society

However, it is worth to mention that in some cases the criteria of aromaticity may show different trends. An interesting disagreement between the magnetic and energetic criteria of aromaticity was found for coronene and isocoronene [52], as presented in Figs. 8 and 9, respectively.

These two compounds are isomers, thus direct comparisons of “whole molecule” properties are allowed. Using energetic criteria it follows that coronene is more aromatic than its isomer since it is more stable by 105 kcal/mol. This is in contradiction to the magnetic susceptibility exaltation parameters which show the opposite picture: isocoronene exceeds coronene by 51.4 cgs ppm. This is, in turn, in line with the HOMA values calculated for the outer and inner envelopes: for isocoronene HOMA(out) = 0.864, HOMA(inn) = 0.982, whereas for coronene they amount to 0.797 and 0.662, respectively. Computation of the ring current density map by the use of the ipsocentric approach [53] explains these results. Isocoronene shows a clear diatropic circulation on the perimeter reinforced by a weak central circulation in the same sense instead of the paratropic central circulation of coronene, see Fig. 8.

The problem of the disagreement between various criteria of aromaticity was first formulated by Katritzky et al. [54] and then discussed in many subsequent papers in the past two decades [30, 55, 56]. Application of various aromaticity indices to nearly 100 π-electron systems [32] allowed to conclude that aromaticity is a statistically multidimensional phenomenon and various criteria may sometimes present non-equivalent pictures.

### The other indices of aromaticity

In addition to the above-presented characteristics of aromaticity it is necessary to briefly mention some other measures, which do not their origin in the enumerative definition presented in the beginning of this paper. The Bader quantum theory of atoms in molecules (QTAIM) [57–59] allows to analyze charge distribution in molecules. Among many properties accessible by the use of this method, the most useful for structural studies are the charges in the atomic basins and properties in the critical point of bonds and rings. The critical points are characterized by the local extreme of electron density, being a minimum charge density in direction of the bond and maximum in directions perpendicular to the bond (a saddle point). This is the so-called the bond critical point (BCP), moreover, the ring critical point (RCP) can also be determined [60]. In addition, it is also possible to compute the density of electron energy (as a whole and also as its potential and kinetic components) in the critical point. It was shown that for polycyclic benzenoid hydrocarbons the QTAIM parameters in RCP *i.e.* charge, total, kinetic and potential energies very well correlate with HOMA (correlation coefficient, *cc*, always better than 0.98) and with NICS(0) with *cc* = 0.909 [61].

QTAIM also allows to describe ellipticity of a bond in its BCP. It is known that the more double is the bond, the higher is its ellipticity. Thus the next aromaticity parameter based on elipticity, EL was proposed [62] which successfully correlated with other aromaticity indices like HOMA, EN, GEO, PDI [63] FLU [64] and NICS’s. Similar approach was earlier presented [65], although not so well documented.

There are several aromaticity descriptors based directly on atomic charges. For example, FLU (aromatic fluctuation index) [64] describes the fluctuation of electronic charge between adjacent atoms in a given ring. Its good correlation with other aromaticity indices as HOMA, EN, GEO, NICS’s and PDI allowed to accept it as a valuable measure of the aromatic character. PDI (*para*-delocalization index) is defined [63] as the average of all the Bader delocalization indices between the *para*-related atoms in six-membered rings. A good review on various aromaticity indices based on atomic charges is presented in a paper of Bultinck [66] which shows their mutual intercorrelations.

Therefore, comparison with the traditional aromaticity indices as well as with some more recently developed ones should be considered as a rule in establishing of any new aromaticity measure.

## Conclusions

In summary, the critical discussion of aromaticity presented above clearly indicates that it is not a single property of chemical compounds, and hence none of criterion alone is sufficient to unequivocally characterize it. Thus, only the multidimensional view on aromaticity-related chemical properties of a given compound can be reliable. As already stated Tetrahedron Report 520 [4], aromatic compounds are only those which fulfil all criteria (i)–(v) presented in the definition, whereas those compounds that fulfil only some of them are described as partly aromatic compounds.

## Electronic supplementary material

Below is the link to the electronic supplementary material.

Supplementary material 1 (pdf 419 kb)

## Abbreviations

ASE: Aromatic stabilization energy
BCP: Bond critical point
BE: Bond energy
*cc*: Correlation coefficient
EL: Aromaticity index based on ellipticity of bonds
FLU: Aromatic fluctuation index
HOMA: Harmonic oscillator model of aromaticity
NICS: Nucleus independent chemical shifts
NMR: Nuclear magnetic resonance
PDI: *Para*-delocalization index
QTAIM: Quantum theory of atoms in molecules
RCP: Ring critical point
RE: Resonance energy
