# Supplementary material for: Aromaticity: what does it mean?
Source: ChemTexts. 2015 Jun 10;1(3):12. doi: 10.1007/s40828-015-0012-2 (PMC6313370; doi:10.1007/s40828-015-0012-2)
Supplement: Supplementary file 1 — Supplementary material 1 (pdf 419 kb) [file 40828_2015_12_MOESM1_ESM.pdf]

Co to jest aromatyczność?[#]

T.M. Krygowski[a] and H. Szatylowicz[b]

[a] Department of Chemistry, Warsaw University, Pasteura 1, 02-093 Warsaw, Poland. Email: *tmkryg@chem.uw.edu.pl*

[b] Faculty of Chemistry, Warsaw University of Technology, Noakowskiego 3, 00-664 Warsaw, Poland. E-mail: *halina@ch.pw.edu.pl*

In memory of our friends and coworkers Professors Alan R. Katritzky and Paul von Rague Schleyer.

Abstrakt

Termin aromatyczność/aromatyczny jest jednym z najbardziej użytecznych i popularnych pojęć w chemii organicznej i pokrewnych dziedzinach. Jest to jednak pojęcie niejednoznaczne, jego definicja jest enumeryczna. Cząsteczki danego cyklicznego lub policyklicznego związku chemicznego są aromatyczne gdy charakteryzuje je podwyższona trwałość (kryterium energetyczne), małe zróżnicowanie długości wiązań (kryterium geometryczne), zewnętrzne pole magnetyczne wzbudza w nich diatropowy prąd kołowy (kryterium magnetyczne), zaś w reakcjach układy te zachowują strukturę π-elektronową. Trwałość, jako cecha aromatyczności, omawiana jest na przykładzie energii rezonansu (RE) i w bardziej nowoczesnym ujęciu – energii stabilizacji aromatycznej (ASE). Kryterium geometryczne jest ilustrowane poprzez indeks HOMA (Harmonic Oscillator Model of Aromaticity). Magnetyczne kryteria przedstawione są jako stosowane zarówno do opisu aromatyczności poszczególnych pierścieni jak i charakteryzujące całe cząsteczki. W pierwszym przypadku (indeks lokalny) można wykorzystać indeks NICS (nucleus independent chemical shifts) czy też przesunięcia chemiczne protonów w spektroskopii ^{1}H NMR (nuclear magnetic resonance), zaś przykładem indeksu globalnego jest egzaltacja podatności magnetycznej. W tabelach podane zostały dane umożliwiające porównanie wyżej wymienionych indeksów dla wybranych związków homo- i heterocyklicznych. Przedstawiono również przykłady zarówno zgodnej jak i rozbieżnej oceny aromatyczności układu (cząsteczki) przez różne indeksy (kryteria) delokalizacji π-elektronowej.

Słowa kluczowe: aromaticity, HOMA, NICS, resonance energy, pi-electron delocalization, ring current

Wykaz skrótów

ASE – energia stabilizacji aromatycznej (aromatic stabilization energy)

BCP – krytyczny punkt wiązania (bond critical point)

BE – energia wiązania (bond energy)

cc – współczynnik korelacji (correlation coefficient)

EL – indeks aromatyczności wykorzystujący eliptyczność wiązania
(aromaticity index based on ellipticity of bonds)

FLU – indeks fluktuacji gęstości ładunku (aromatic fluctuation index)

HOMA – model oscylatora harmonicznego aromatyczności
(harmonic oscillator model of aromaticity)

NICS – przesunięcie chemiczne niezależne od jądra (nucleus independent chemical shifts)

NMR – magnetyczne rezonans jądrowy (nuclear magnetic resonance)

PDI – indeks delokalizacji *para* (*para*-delocalization index)

QTAIM – kwantowa teoria atomy w cząsteczce (quantum theory of atoms in molecules)

RCP – punkt krytyczny pierścienia (ring critical point)

RE – energia rezonansu (resonance energy)

Wprowadzenie

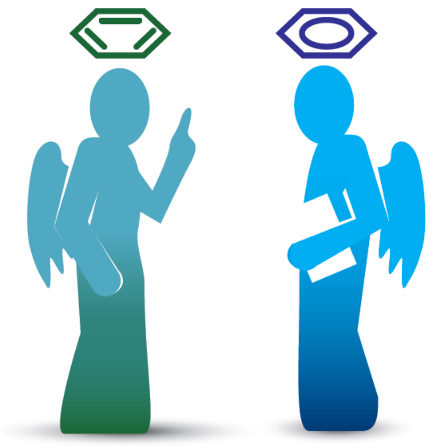

Rys. 1. Aromatyczność jako zagadnienie delokalizacji elektronów π w układach cyklicznych

Aromatyczność jest bardzo często wykorzystywanym terminem w chemii organicznej i naukach pokrewnych. Codziennie ukazuje się ponad 30 prac, w których aromatic/aromaticity znajduje się w tytule, abstrakcie lub w słowach kluczowych [1]. Rozliczne związki organiczne są aromatyczne lub zawierają aromatyczny fragment. Ich aromatyczność jest określana poprzez zespół właściwości chemicznych i fizykochemicznych π-elektronowych układów cyklicznych i policyklicznych. Właściwości te wiążą się z delokalizacją elektronów π, por. Rys.1, i definiują układ aromatyczny jako taki, który [2,3,4]:

(i) jest związkiem cyklicznym lub policyklicznym;

(ii) jest trwalszy niż jego acykliczny analog (olefiny);

(iii) ma wyrównane długości wiązań, bliskie wartości średniej;

(iv) pod wpływem zewnętrznego pola magnetycznego wzbudzany jest prąd kołowy powodujący wzrost podatności diamagnetycznej i diatropowe (w kierunku niższego pola) przesunięcia chemiczne egzocyklicznych protonów w widmach spektroskopowych ^{1}HNMR,

(v) układy aromatyczne wykazują skłonność do zachodzenia reakcji z zachowaniem struktury π-elektronowej (preferowane jest podstawienie a nie addycja)

Powyższe właściwości stanowią kryteria aromatyczności, które w pewnym stopniu można wyrazić ilościowo jako tzw. indeksy aromatyczności.

Energetyczne miary aromatyczności

Początki chemicznego znaczenia pojęcia aromatyczność sięgają prac połowy XIX w., gdy F.A. Kekule von Stradonitz zauważył w swoim podręczniku [5], że związki aromatyczne (głównie benzen) w porównaniu do olefin wykazują oporność wchodzenia w reakcje chemiczne. Dopiero w 1933 Pauling [6] określił ilościowo trwałość układów π-elektronowych wprowadzając pojęcie energii rezonansu (RE) jako energii, o jaką układ aromatyczny jest trwalszy od jego acyklicznego analogu. RE oparta jest na pomiarze kalorymetrycznym benzenu oraz wartościach energii wiązań C=C, C-C i CH, również wyznaczonych kalorymetrycznie. Obliczona RE benzenu wynosiła 36 kcal/mol. Trzy lata później wyznaczono RE benzenu, także w oparciu o pomiary kalorymetryczne [7], na podstawie reakcji uwodornienia benzenu i cykloheksenu [8], uzyskując podobną wartość.

Współcześnie termin RE jest raczej zarzucony na korzyść bardziej precyzyjnych definicji energii stabilizacji aromatycznej (ASE, aromatic stabilization energy), które mogą być określane na różnych poziomach dokładności jako reakcje izodesmiczne [9**]** albo bardziej precyzyjne reakcje homodesmotyczne [10,11]**.** Te ostatnie są zdefiniowane jako wirtualne reakcje, w których po obu stronach znajduje się taka sama liczba wiązań CH i takie same liczby atomów w odpowiednich stanach hybrydyzacji. Schemat 1 oraz Tablica 1 przedstawiają dane strukturalne i liczbowe takiej przykładowej reakcji.

Schemat 1 Przykład reakcja homodesmotycznej

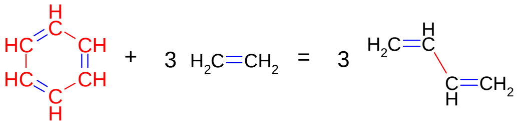

Tablica 1. Typy wiązań homodesmotycznej reakcji przedstawionej na Schemacie 1

Substraty	Liczba wiązań	Produkty	Liczba wiązań
$C^{(sp2)} = C^{(sp2)}$	6	$C^{(sp2)} = C^{(sp2)}$	6
$C^{(sp2)} – C^{(sp2)}$	3	$C^{(sp2)} – C^{(sp2)}$	3
$C^{(sp2)}$ – H	18	$C^{(sp2)}$ – H	18

$C_6H_6 + 3\ CH_2CH_2 = 3$ *trans* $CH_2CHCHCH_2$ ASE = 23.2 kcal/mol

$C_6H_6 + 3\ CH_2CH_2 = 3$ *cis* $CH_2CHCHCH_2$ ASE = 33.6 kcal/mol

Bardziej szczegółowo zagadnienia te są omówione w artykule przeglądowym Cyrańskiego [12], w którym pokazano jak kluczowy charakter ma wybór odpowiednich układów odniesienia. Tablica 2 przedstawia dane dotyczące reakcji izodesmicznych i homodesmotycznych dla różnych układów odniesienia i różnych poziomów obliczeń.

Tablica 2. Energie stabilizacji[(a)] reakcji ISODESMICZNYCH i HOMODESMOTYCZNYCH

$C_6H_6 + 6\ CH_4 = 3\ CH_3CH_3 + 3\ CH_2CH_2$

$C_6H_6 + 3\ CH_2CH_2 = CH_2CHCHCH_2$

$C_6H_6 + 3\ CH_2CHCHCH_2 = 3\ CH_2CHCHCHCHCH_2$

Poziom obliczeń	ASE / kcal/mol
MP2/RHF/SKB(d)	74.7
MP2/6-31G//6-31G*	67.2
HF/6-31G*	58.2
MP4/SDTQ/6-31G**//MP2(full)/6-31G**	23.9
RMP2/6-311G**	28.0
B3LYP/6-311+G*	22.2
6-31G* (SCF)	24.7
MP4/SDTQ/6-31G**//MP2(full)/6-31G**	20.3
6-31G* (SCF)	23.4

(a) Dane z pracy [12]

Jak widzimy, wartości ASE znacząco zależą od wyboru obu tych czynników. Niezależnie od wskazanych ograniczeń, podejście typu ASE daje się zastosować głównie do węglowodorów π-elektronowych. W odniesieniu do układów heterocyklicznych, są one trudniejsze do zastosowania i mniej efektywne. W niektórych przypadkach – na przykład w odniesieniu do izomerów lub tautomerów, można stosować bezpośrednie porównanie energii całkowitej. Uważa się, że kryterium energetyczne jest najważniejsze dla oceny aromatyczności układów π-elektronowych. Jednakże pojawia się problem w przypadku cząsteczek wielopierścieniowych – poszczególne pierścienie mogą różnić się aromatycznością. Dobrym przykładem jest fenantren, w którym centralny pierścień jest bardziej reaktywny niż dwa pozostałe. Objawia się to tym, że w położeniach 9 i 10 fenantrenu zachodzą reakcje addycji, podczas gdy pozycje w pozostałych pierścieniach są inertne, a jeżeli już, to zachodzą reakcje substytucji. Nie ma łatwych bezpośrednich metod wyznaczenia energii pojedynczych pierścieni w układach wielopierścieniowych, chociaż są

próby szacowania takich energii w oparciu o ich geometrię (długości wiązań) [13,14,15], porównaj artykuł przeglądowy [16].

Geometryczne kryterium aromatyczności – indeks HOMA

Następne ważne kryterium aromatyczności opiera się na geometrii cząsteczek, a dokładniej na długościach wiązań. Im bardziej aromatyczny jest układ tym bardziej wyrównane są długości wiązań. Ilościowo ujął to Julg i współ. [17] definiując indeks aromatyczności *A*, będący znormalizowaną funkcją wariancji długości wiązań obwodu cząsteczki. Niestety parametr ten mógł być stosowany tylko do układów karbocyklicznych, ponieważ nie ma możliwości obliczania wartości średnich długości wiązań CC i takich, które zawierają heteroatomy. Konieczne było poprawienie modelu. Zrobiono to w ten sposób, że długość średnią wiązania w wyrażeniu na wariancję zastąpiono przez długość optymalną, równanie (1) R_{opt}, hipotetyczną wielkość jaką wiązanie danego typu miało by w układzie idealnie aromatycznym [18,19]. Indeks ten nazwano HOMA (od harmonic oscillator model of aromaticity) i jest zdefiniowany równaniem:

$$\mathrm{HOMA} = 1 - \frac{\alpha_j}{n}\sum_{i}^{n}(R_{\mathrm{opt},j} - R_{j,i})^2 \tag{1}$$

gdzie α_j oraz $R_{\mathrm{opt},j}$ są parametrami zależnymi od rodzaju wiązania (np. *j* może być wiązaniem CC, CN, CO, CP, CS, NN, NO, itd.). Parametr α (stała normalizacyjna) jest wyznaczany z wykorzystaniem optymalnych (R_{opt}) i doświadczalnych referencyjnych długości wiązań pojedynczych (R_{s}) oraz podwójnych (R_{d}). R_{opt} wyznacza się z warunku, aby energia (obliczana w ramach modelu oscylatora harmonicznego) jego rozciągnięcia do długości wiązania pojedynczego była równa energii kompresji do długości wiązania podwójnego. Natomiast $R_{j,i}$ są długościami wiązań typu *j* dla *i*=*n* wiązań wziętych do obliczeń. Tablica 3 przedstawia dane niezbędne do obliczania parametru HOMA dla układów z wiązaniami w niej podanymi [16]. Konieczna jest tylko wiarygodna geometria interesującego nas układu.

Tablica 3. Długości wiązań referencyjnych R_s i R_d, oraz wartości parametrów R_{opt} i α do obliczeń indeksu HOMA

Typ wiązania	R_s /Å	R_d /Å	R_{opt} /Å	α	Literatura
BB[(a)]	1.6474	1.5260	1.5665	244.147	[20]
BB[w (a)]	1.6474	1.5260	1.5693	250.544	[20]
BC[exp (b)]<br>BC[theo (b)]<br>BC[theo/w (b)]	1.5472<br>1.5542<br>1.5542	1.3616<br>1.3796<br>1.3766	1.4235<br>1.4378<br>1.4386	104.507<br>118.009<br>118.618	[21]<br>[21]<br>[21]
BN[(c)]	1.564	1.363	1.402	72.03	[22]
CC[(d)]	1.467	1.349	1.388	257.7	[23]
CN[(e)]	1.465	1.269	1.334	93.52	[23]
CO[(f)]	1.367	1.217	1.265	157.38	[23]
CP[(g)]	1.814	1.640	1.698	118.91	[23]
CS[(h)]	1.807	1.611	1.677	94.09	[23]
CSe[(i)]	1.959	1.7591	1.8217	84.9144	[24]
NN[(j)]	1.420	1.254	1.309	130.33	[23]
NO[(k)]	1.415	1.164	1.248	57.21	[23]

Wykorzystane układy odniesienia: (a) H_2B-BH_2 i HB=BH; (b) H_3C-BH_2 i H_2C=BH; (c) H_3B-NH_3 i (*iso*Pr)$_2$N=B=C(SiMe$_3$)$_2$, H_3B-NH_3 i H_2B=NH_2; (d) buta-1,3-diene; (e) H_2N-CH_3 i HN=CH_2; (f) HCOOH monomer; (g) H_2C=P-CH_3; (h) S(CH_3)$_2$ i H_2C=S; (i) H_3C-SeH i H_2C=Se; (j) (CH_3)$_2$C=N-N(CH_3)$_2$ i H_3C-N=N-CH_3; (k) CH_3-O-N=O.

Wyrażenie (1) można analitycznie przekształcić [25] do postaci dwóch członów, GEO i EN opisanych poniższymi równaniami:

$$\mathrm{HOMA} = 1 - \frac{1}{n}\sum_i \alpha\left(R_{\mathrm{opt}} - R_i)\right)^2 = 1 - \mathrm{EN} - \mathrm{GEO} \quad (2)$$

gdzie

$$\mathrm{GEO} = \frac{1}{n}\sum_i \alpha\left(R_{\mathrm{av}} - R_i)\right)^2 \quad (3)$$

$$\mathrm{EN} = \alpha\left(R_{\mathrm{opt}} - R_{\mathrm{av}}\right)^2, \quad (4)$$

które przedstawiają dwie składowe decydujące o obniżeniu aromatyczności, czyli zmniejszeniu wartości HOMA. Człon GEO (3) opisuje stopień alternacji długości wiązań – im jest większy, tym aromatyczność (czyli HOMA) jest mniejsza. Człon ten jest równoważny indeksowi aromatyczności Julga [17]. Człon EN (4) określa, o ile średnia długość wiązań

układu jest większa (lub mniejsza) od wartości średniej. Im większa ta różnica, tym układ jest mniej aromatyczny (jest niższa wartość HOMA). Jest to skutkiem faktu, że oba te człony odejmuje się w równaniu (2) od jedności.

Rozważmy zastosowania równań (1)–(4) do opisu aromatyczności fenantrenu i trójfenylenu [26], przedstawionych na Rys. 2.

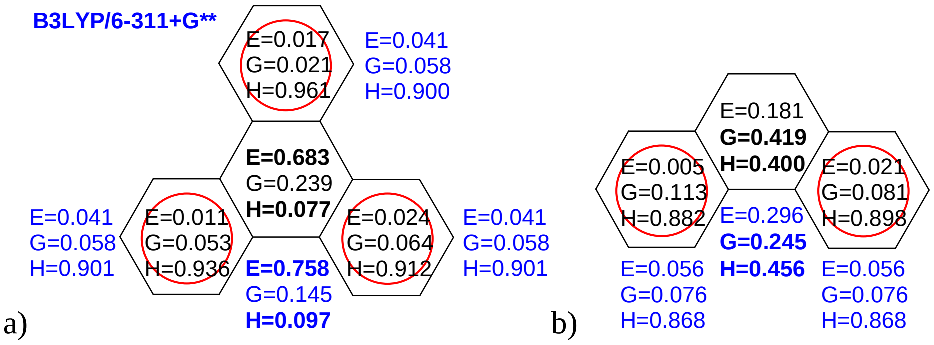

Rys. 2 Zależność charakteru aromatycznego pierścieni benzenowych od ich otoczenia topologicznego w cząsteczkach węglowodorów benzenoidowych (a) trójfenylenu i (b) fenantrenu [26]; porównanie wartości pochodzących z obliczeń z wartościami wyznaczonymi na podstawie danych eksperymentalnych

Można zauważyć kilka ciekawych aspektów. Po pierwsze, wartości eksperymentalne nie spełniają warunków symetrii, gdyż w komórce elementarnej badanych kryształów cząsteczki te znajdowały się w położeniu ogólnym komórki elementarnej. Oznacza to iż nie mogły mieć narzuconej symetrii i w konsekwencji oddziaływanie „formalnie symetrycznych" części tych cząsteczek miały odmienne otoczenie w krysztale, a więc także odmienne oddziaływania z sąsiadami, a co za tym idzie odmienne deformacje [27]. Po drugie zauważmy, że wartości parametrów aromatyczności uzyskane z danych geometrycznych obliczonych metodą B3LYP/6-311+G** nie różnią się w istotny sposób od tych uzyskanych z danych doświadczalnych. Po trzecie zauważmy, że pierścienie zewnętrzne w obu cząsteczkach mają wysokie wartości HOMA, ~0.9 lub wyższe, podczas gdy pierścienie centralne mają obniżone wartości HOMA. Jeżeli spojrzymy na wartości EN i GEO dla pierścieni centralnych to zauważymy, że w przypadku trójfenylenu wartość EN jest bardzo wysoka, ~0.7, podczas gdy GEO jest niskie ~0.2. Gdy oba czynniki odejmiemy od 1 w równaniu (2), uzyskamy HOMA ~0.1 zaś decydującym czynnikiem obniżenia aromatyczności są tu wydłużone wiązania w pierścieniu centralnym. Odmienna jest sytuacja w przypadku fenantrenu, gdzie wartość GEO jest duża, ~0.35, EN jest też duże ale mniejsze niż GEO (~0.25) i stąd HOMA ~0.4. Główną przyczyną obniżenia aromatyczności jest tu wzrost alternacji długości wiązań. Według klasyfikacji Clara [28,29] pierścień centralny

w trójfenylenie jest “pusty” tj. zawiera mało elektronów π, natomiast pierścień centralny w fenantrenie jest pierścieniem nienasyconym, tj. zawierającym wiązanie podwójne, a więc o większej alternacji długości wiązań. Obliczenia energii tych pierścieni wg metody podanej w pracy [30] pokazały, że oba centralne pierścienie w fenantrenie i trójfenylenie mają mniejszą zawartość energii (BE = 669.4 i 668.9 kcal/mol, odpowiednio) podczas gdy pierścienie zewnętrzne mają wartości BE w granicach 715.6 – 725.2 kcal/mol. Jak widzimy, HOMA i BE są w zgodzie z jakościową koncepcją klasyfikacji węglowodorów benzenoidowych Clara. Warto też zauważyć, że składowe EN i GEO indeksu HOMA nadają obniżeniu aromatyczności pierścieni centralnych ważny aspekt strukturalny.

Wartości indeksu HOMA oraz EN i GEO wybranych cząsteczek związków homo- i heterocyklicznych zostały przedstawione w Tablicy 4.

Tablica 4. Wartości HOMA EN i GEO wybranych związków homo- i heterocyklicznych

	HOMA	EN	GEO	Reference
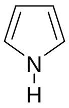 Pirol	0.86			[31]
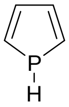 Fosfol	0.236			[32]
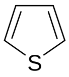 Tiofen	0.75	0.04	0.21	[33]
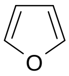 Furan	0.20	0.20	0.60	[33]
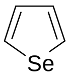 Selenofen	0.72	0.03	0.25	[33]
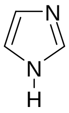 Imidazol	0.88			[31]
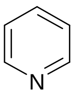 Pirydyna	0.998	-0.009	0.011	[34]
Benzen	0.979	0.021	0.000	[26]
Naftalen	0.802	0.077	0.121	[26]
Cyklopentadien	-0.778			[35]
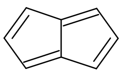 Pentalen	-0.381			[36]

Magnetyczne indeksy aromatyczności

Ważnym i łatwo dostępnym, kryterium aromatyczności są właściwości magnetyczne cząsteczek. Od dawna wiadomo, na podstawie wyników spektroskopii ^{1}H NMR, że egzocykliczne protony są odsłaniane, czyli wartości ich przesunięcia chemicznego są większe niż w przypadku protonów olefinowych, odpowiednio ~7ppm i ~5 ppm [37]. Rysunek 3 ilustruje to na przykładzie benzenu.

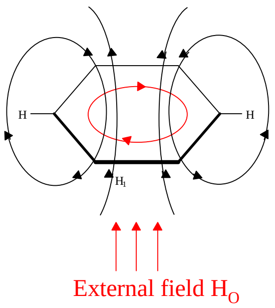

Rys. 3 Zewnętrzne pole magnetyczne indukuje prąd pierścieniowy, powodujący charakterystyczne wartości przesunięć chemicznych protonów w spektroskopii ^{1}HNMR. Przedruk za zgodą z [12]. Copyright 2005 American Chemical Society

Jednakże wartości przesunięć wyraźnie zależą od położenia protonu w cząsteczce, co przedstawiają dane w Tablicy 5 na przykładzie wartości przesunięć protonów w fenantrenie, zmierzonych CD_3Cl [38].

Tablica 5. Wartości przesunięć chemicznych protonów fenantrenu [38]

	Położenie protonu	Przesunięcie chemiczne /ppm
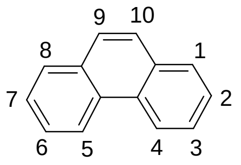	1	7.901
	2	7.606
	3	7.666
	4,5	8.702
	9,10	7.751

Zatem można uznać, że przesunięcia chemiczne w spektroskopii ^{1}H NMR mogą służyć w pewnym stopniu jako wielkości charakteryzujące lokalną aromatyczność. Warto jednak pamiętać, że na wartości przesunięć chemicznych wpływają także warunki pomiaru: czy to

jest ciało stałe lub jaki jest rodzaj rozpuszczalnika, gdy pomiary są rejestrowane w roztworach.

Innym lokalnym parametrem charakteryzującym aromatyczność danego pierścienia jest wprowadzony przez Schleyera i współ. czysto teoretyczny indeks NICS (skrót od nucleus independent chemical shift) [39,40], który szybko stał się bardzo popularny. NICS jest zdefiniowany jako ujemna wartość przesłaniania mierzona (obliczana) w środku pierścienia [NICS(0)], jeden Å powyżej środka [NICS(1)] oraz jako prostopadła składowa tensora przesłaniania na wysokości 1 Å [NICS$(1)_{zz}$]. Tablica 6 przedstawia [39] wartości NICS kilku wybranych układów. Im bardziej ujemna wartość NICS, tym wyższa jest aromatyczność pierścienia dla którego została określona. Jak łatwo można zauważyć występują nieco niespodziewane rozbieżności. Pierścienie w naftalenie są bardziej aromatyczne niż w benzenie, co jest w sprzeczności z oceną energetyczną aromatyczności. Podobnie pirol, tiofen i furan są sklasyfikowane jako bardziej aromatyczne niż benzen, co też nie odpowiada doświadczeniu chemików względem tych połączeń. Trzeba bowiem wyraźnie zaznaczyć, że właściwości magnetyczne (NICS-y) zależą od powierzchni badanej cząsteczki.

Tablica 6. Wartości magnetycznych indeksów aromatyczności: egzaltacja podatności magnetycznej (Λ) i NICS(0) wybranych homo- i heterocyklicznych związków

	Λ /cgs·ppm	Literatura Λ	NICS(0) /ppm	Literatura NICS(0)
Pyrol	-6.5	[32]	−15.1	[39]
Fosfol	-1.7	[32]	-5.35	[40]
Tiofen	-7.0	[32]	−13.6	[39]
Furan	-2.9	[32]	−12.3	[39]
Benzen	-10.47	[41]	−9.7	[39]
Naftalen	-20.98	[41]	−9.9	[39]
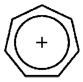 Tropylium	-13.81	[41]	−7.6	[39]
Cyklopentadien	-2.4	[39]	−3.2	[39]
Cykloheksan	-0.7	[39]	−2.2	[39]
Pentalen	34.59	[41]	18.1	[39]
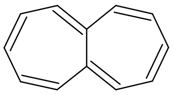 Heptalen	76.6	[39]	22.7	[39]
Cyklobutadien	17.20	[41]	27.6	[39]

Oprócz wspomnianych powyżej lokalnych magnetycznych parametrów aromatyczności są znane dwie ważne charakterystyki odnoszące się do całych cząsteczek, czyli tzw. indeksy globalne. Są to: anizotropia podatności magnetycznej $\Delta\chi$ [42] (5) oraz egzaltacja podatności magnetycznej Λ [43] (6).

$$\Delta\chi = \chi_{cc} - \tfrac{1}{2}(\chi_{aa} + \chi_{bb}) \quad (5)$$

i

$$\Lambda = \chi_{M} - \chi_{M'} \quad (6)$$

gdzie χ jest elementem diagonalnym tensora podatności magnetycznej, natomiast *c* jest kierunkiem prostopadłym do płaszczyzny cząsteczki. W obu przypadkach charakterystyki te są wielkościami względnymi. Pierwsza z nich odnosi się do średniej wartości podatności magnetycznej w płaszczyźnie *ab* (tj. cząsteczki), zaś druga odnosi się do podatności magnetycznej cząsteczki nienasyconej (analog olefinowy), a więc sytuacja podobna jak z wyznaczaniem energii rezonansu RE lub ASE. Ostatnio znaleziono relację między sumą $NICS(1)_{zz}$ składowych pierścieni szeregu węglowodorów aromatycznych i antyaromatycznych a ich wartościami egzaltacji podatności magnetycznej [41].

Wpływ wewnątrz- i międzycząsteczkowych oddziaływań na aromatyczność pierścienia

Innym ciekawym zagadnieniem jest wpływ różnorodnych oddziaływań, podstawników i/lub międzycząsteczkowego wiązania wodorowego, na aromatyczność pierścienia. Dobrym przykładem jest zmienność aromatyczności pierścienia benzenowego pochodnych fenolu i jonów fenolanowych. Rozważmy hipotetyczną sytuację, gdy do grupy hydroksylowej pochodnej fenolu lub do tlenu w pochodnej fenolanowej zbliżają się odpowiednio F^- albo HF (ilustruje to Rys. 4).

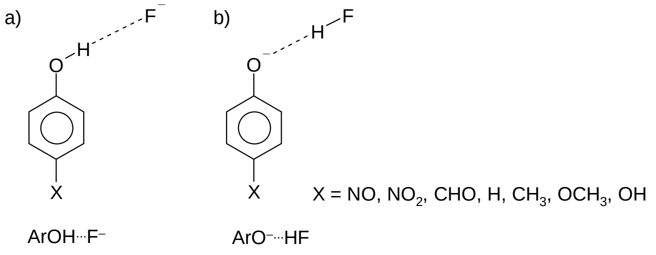

Rys. 4 Strukturalny schemat modelu obliczeniowego wpływu podstawnika oraz wiązania wodorowego na aromatyczność pierścienia fenylowego

W obu przypadkach powstają kompleksy z utworzeniem wiązania wodorowego, w wyniku czego zmienia się geometria pierścienia [44]. Obliczone wartości HOMA pokazują jak duży jest wpływ wiązania wodorowego, którego moc jest monitorowana przez długość wiązania CO [45,46], na aromatyczność pierścienia (Rys. 5).

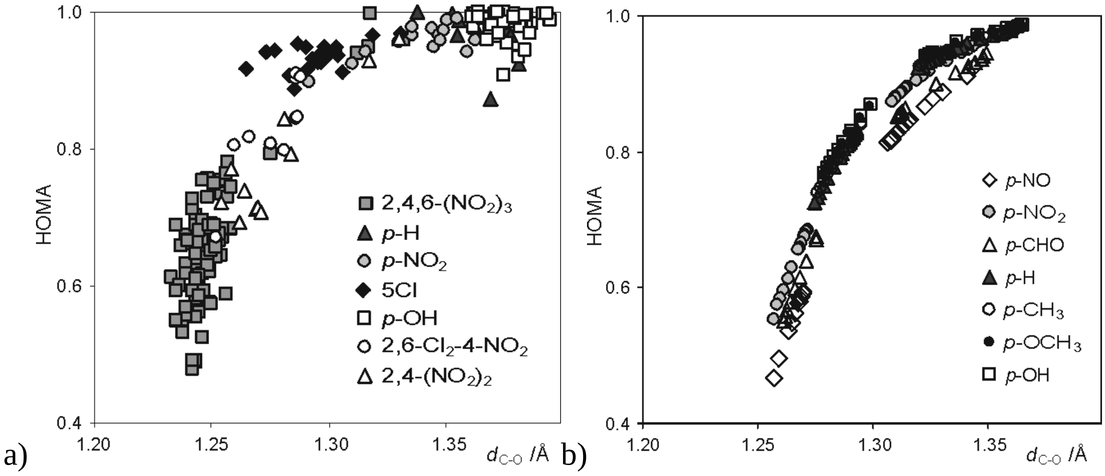

Rys. 5 Zależność aromatyczności pierścienia fenylowego (HOMA) od siły wiązania wodorowego. Wyniki (a) X-ray (CSD, 664 geometrii) oraz (b) B3LYP/6-311+G**. Przedruk za zgodą z [45,46]. Copyright 2004 and 2005 American Chemical Society

Co więcej, wykorzystanie parametrów geometrycznych struktur kompleksów różnie podstawionych pochodnych fenolu i fenolanów, uwikłanych w wiązanie wodorowe, pokazało pełną jakościową zgodność „obrazów" wpływu oddziaływań na aromatyczność pierścienia otrzymanych z obliczeń oraz uzyskanych na podstawie danych doświadczalnych [45,46].

Jednym z ważniejszych problemów w chemii organicznej jest wpływ podstawników na właściwości badanych układów. Klasyczne podejście do efektu podstawnikowego jest związane z podstawionymi pochodnymi benzenu i opisane przez fundamentalną teorię wprowadzoną przez L.P. Hammetta [47], zaś nowszy przegląd można znaleźć w [48]. Zastosowanie idei Hammetta w przypadku *para* podstawionych pochodnych fenolu, fenolanów i ich równowagowych kompleksów z wiązaniem wodorowym zostało przedstawione na Rys. 6.

a) 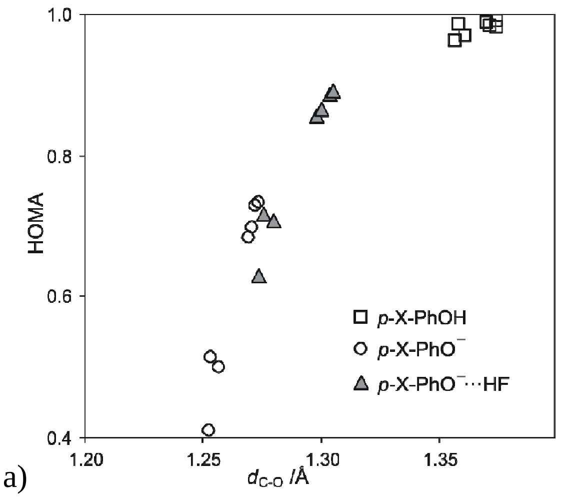

b) 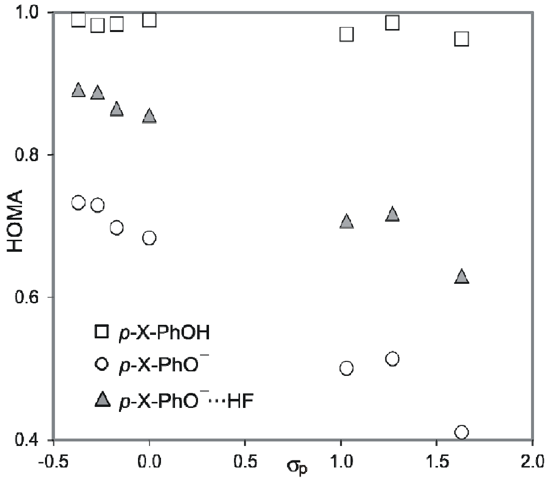

Rys. 6 Zależność aromatyczności pierścienia fenylowego (HOMA) od (a) długości wiązania C-O, $d_{\text{C-O}}$, oraz (b) stałej podstawnika σ_p (w przypadku podstawników elektrono-akceptorowych użyto σ_p^-) *para* podstawionych pochodnych fenolu, fenolanów i ich równowagowych kompleksów z wiązaniem wodorowym (*p*-X-PhO⁻···HF). Część (a) przedrukowana za zgodą z [46]. Copyright 2005 American Chemical Society

a) 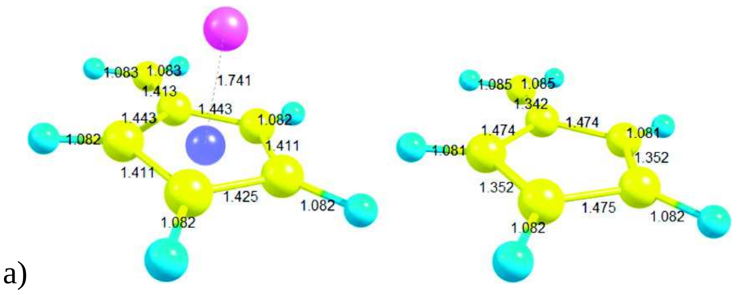

b) 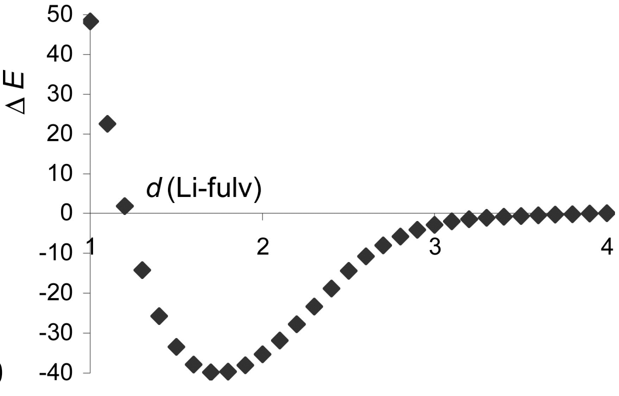

Rys. 7 (a) Swobodna cząsteczka fulwenu oraz obliczona struktura jego komplesu z Li [B3LYP/6-311++G(d,p)] (b) Względna energia, ΔE (kcal/mol), kompleksu fulwenu z Li w funkcji odległości Li od płaszczyzny fulwenu, *d*(Li-fulv) (Å). Przedruk za zgodą z [49]. Copyright 2010 American Chemical Society

Należy wspomnieć, że w wielu przypadkach ma miejsce zgodna ocena aromatyczności na podstawie HOMA, NICS i energetyki badanych układów. Dobrym przykładem jest powstawanie kompleksu fulwenu z atomem litu [49]. Rysunek 7 przedstawia zarówno

schemat układu jak i krzywą zależności energii kompleksu od odległości atomu Li od płaszczyzny pierścienia.

Fulwen jest znanym nienaprzemiennym węglowodorem π-elektronowym [50,51]. Przybliżanie atomu litu prowadzi do częściowego przeniesienia elektronu z atomu Li na układ π-elektronowy fulwenu. Powoduje to zwiększenie stabilności układu o ok. 40 kcal/mol oraz wzrost charakteru aromatycznego fulwenu – od HOMA ~-0.3 dla swobodnej cząsteczki do ~0.6 dla kompleksu równowagowego. Odpowiednie wartości NICS wynoszą 0.94 i -11.15 [49], czyli również potwierdzają wzrost aromatyczności pierścienia.

Wielowymiarowy charakter aromatyczności

Ważnym aspektem opisu delokalizacji π-elektronowej jest występowanie niezgodności między indeksami aromatyczności w przypadku klasycznych układów. Taka sytuacja ma miejsce w przypadku koronenu i izokoronenu, przedstawionych na Rys. 8 i 9 [52].

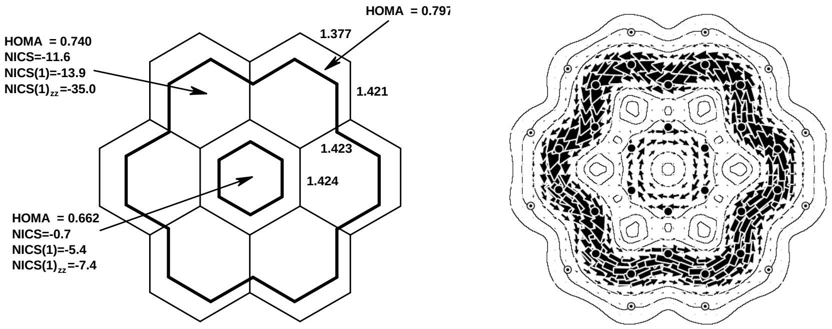

(a) (b)

Rys. 8 (a) Długości wiązań i deskryptory aromatyczności: HOMA, NICS, NICS(1) oraz NICS(1)$_{zz}$ dla fragmentów koronenu. (b) Mapa gęstości prądu π- elektronowego w koronenie. Prądy diatropowe oraz paratropowe zaznaczone są jako odpowiednio przeciwne oraz zgodne z ruchem wskazówek zegara. Przedruk za zgodą z [52]. Copyright 2006 American Chemical Society

Koronen oraz izokoronen są izomerami. Zatem możliwe jest porównanie deskryptorów aromatyczności opisujących całe cząsteczki. Okazuje się, że koronen jest trwalszy od izokoronenu o 105 kcal/mol natomiast podatność magnetyczna wskazuje odwrotną relację. Izokoronen ma wyższą podatność magnetyczną niż koronen o 51.4 cgs·ppm. Tak więc kryterium energetyczne jest sprzeczne z kryterium magnetycznym. Wartości HOMA dla obwiedni zewnętrznej i wewnętrznej izokoronenu wynoszą 0.864 i 0.982, podczas gdy w przypadku koronenu są to wartości 0.797 i 0.662. A więc kryterium

geometryczne jest tutaj zgodne z obrazem magnetycznym. Wytłumaczeniem tej niezgodności jest postać prądów kołowych indukowanych przez zewnętrzne pole magnetyczne. Obliczone metodą ipsocentryczną [53] kierunki prądów kołowych są w obu przypadkach zasadniczo różne (por. Rys. 8 i 9). W przypadku koronenu prąd kołowy w wewnętrznym pierścieniu ma kierunek zgodny z ruchem wskazówek zegara, jest to więc prąd paratropowy, podczas gdy prąd kołowy obwiedni jest diatropowy. A więc w podatności magnetycznej prądy te częściowo się znoszą. Odmienna jest sytuacja w przypadku izokoronenu, gdzie oba prądy są diatropowe i stąd wysoka wartość podatności magnetycznej.

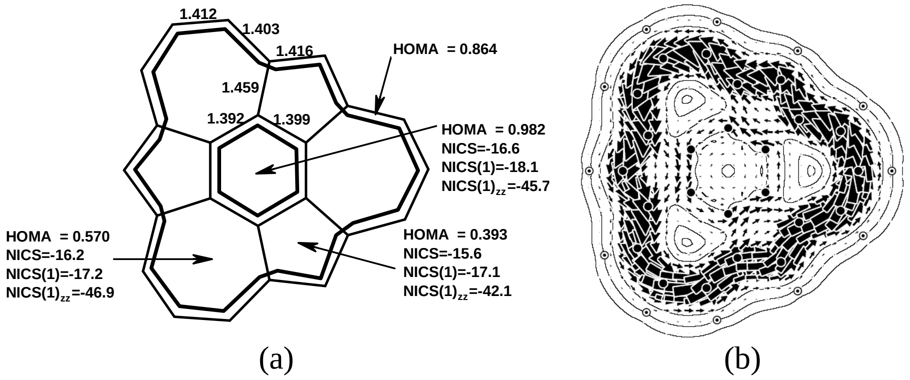

Rys. 9 (a) Długości wiązań i deskryptory aromatyczności: HOMA, NICS, NICS(1) oraz $NICS(1)_{zz}$ dla fragmentów izokoronenu (b) Mapa gęstości prądu π-elektronowego w izokoronenie. Prądy diatropowe oraz paratropowe zaznaczone są jako odpowiednio przeciwne oraz zgodne z ruchem wskazówek zegara. Reprinted with permission from [52]. Copyright 2006 American Chemical Society

Jak widzimy, występuje poważny problem niezgodności oceny aromatyczności opisywanej różnymi deskryptorami aromatyczności. Problem ten zauważył Katritzky i współ. [54], a następnie liczni inni autorzy [30,55,56]. Ostatecznie, na podstawie badań aromatyczności ponad stu układów π-elektronowych z zastosowaniem wielu indeksów aromatyczności uznano, że aromatyczność jest zjawiskiem statystycznie wielowymiarowym i różne kryteria mogą sugerować nierównoważny opis delokalizacji π-elektronowej [32].

Inne indeksy aromatyczności

Oprócz wspomnianych kryteriów aromatyczności i w konsekwencji ilościowych deskryptorów, w ostatnich dwudziestu latach pojawiło się wiele odmiennych sugestii oceny aromatyczności. W dużym stopniu wiąże się to z pojawieniem się kwantowej teorii atomy w cząsteczce (QTAIM, od Quantum Theory Atoms in Molecules) Richarda Badera

[57,58,59], która pozwala na wiarygodne oszacowanie ładunku elektronowego na atomach, właściwości punktów krytycznych wiązań (BCP) i pierścieni (RCP). Wielkości te zostały wykorzystane do oceny aromatyczności [60] w licznych pracach, które ukazały się w ostatnich dwóch dekadach. Okazało się, że wartości ładunku, gęstości całkowitej energii a także energii kinetycznej i potencjalnej w RCP wyznaczone dla szeregu pierścieni w węglowodorach benzenoidowych dobrze korelują z wartościami HOMA [61]. W innym przypadku wykorzystano eliptyczność wiązań i skonstruowano indeks aromatyczności EL [62], dobrze korelujący z innymi indeksami aromatyczności takimi jak: HOMA, EN, GEO, PDI [63], FLU [64] i NICS. Podobny indeks w oparciu o eliptyczności wiązań zaproponował wcześniej Matta i współ. [65]. Dzięki możliwości obliczania ładunków atomowych, zaproponowano indeks FLU, obrazujący fluktuacje gęstości ładunku atomowego pomiędzy sąsiadującymi atomami [64]. FLU koreluje dobrze z innymi indeksami aromatyczności, takimi jak HOMA i NICS-y. Wprowadzono także indeks PDI [63] jako średni parametr delokalizacji pomiędzy atomami będącymi względem siebie w pozycji *para*. Przegląd różnych indeksów aromatyczności opracowanych na podstawie ładunków elektronowych na atomach przedstawił Bultinck [66], pokazując także ich wzajemne korelacje.

Na ogół procedura wprowadzania nowych idei oceny aromatyczności cząsteczek lub poszczególnych pierścieni związana była z porównaniem wartości tych nowych parametrów z tymi, które zostały już wcześniej uznane.

Wnioski

Podsumowując, należy podkreślić fakt, iż pojęcie aromatyczności nie odnosi się do jakiejś pojedynczej właściwości cząsteczki, czyli spełnienie pojedynczego kryterium aromatyczności nie może być decydujące. Wydaje się rozsądnym aby przyjąć, że aromatyczność jest właściwością kolektywną i dopiero spełnienie wszystkich kryteriów (i–v) [4] pozwala na to aby dany związek chemiczny uznać jako w pełni aromatyczny. Natomiast układ spełniający tylko część tych kryteriów należy uznać jako częściowo aromatyczny.

Podziękowania

Autorzy są bardzo wdzięczni Jarkowi Kucharczykowi za przygotowanie graficznego „obrazu aromatyczności”, przedstawionego na Rysunku 1. T.M.K. dziękuje Uniwersytetowi Warszawskiemu, zaś H.S. – Politechnice Warszawskiej, za wsparcie tej pracy.

Literatura

1. ISI Web of Science, retrieved in December 2014
2. Elvidge JA, Jackman LM (1961) J Chem Soc 859-866
3. Sondheimer F (1964) Pure Appl Chem 7:363-388
4. Krygowski TM, Cyrański MK, Czarnocki Z, Haefelinger G, Katritzky AR (2000) TETRAHEDRON REPORT 520, Tetrahedron 56:1783-1796
5. Kekule FA (1866) Lehrbuch der organischen Chemie, F. Enke Verlag, Erlangen
6. Pauling L, Sherman J (1933) J. Chem. Phys. 1:606-617
7. Kistiakowsky GB, Ruhoff JR, Smith HA, Vaughan WE (1936) J Am Chem Soc 58:146-153
8. Pauling L, The Nature of Chemical Bond, Cornell Univ. Press, Ithaca, 1960, p.193
9. Hehre WJ, Ditchfield R, Radom L, Pople JA (1970) J Am Chem Soc 92:4796-4801
10. Hehre WJ, McIver RT, Pople JA, Schleyer PvR (1974) J Am Chem Soc 96:7162-7163
11. Radom L (1974) J Chem Soc, Chem Commun 403-404

12 Cyranski MK (2005) Chem Rev 105:3773-3811

13. Krygowski TM, Ciesielski A, Bird CW, Kotschy A (1995) J Chem Inf Comput Sci 35:203-210
14. Howard ST, Cyranski MK, Stolarczyk LZ (2001) Chem Comm 197-198
15. Cyranski MK, Howard ST, Chodkiewicz ML (2004) Chem Comm 10:2458-2459
16. Krygowski TM, Szatyłowicz H, Stasyuk OA, Dominikowska J, Palusiak M (2014) Chem Rev 114:6383-6422
17. Julg A, Francois P (1967) Theor Chim Acta 7:249-259
18. Kruszewski J, Krygowski TM (1972) Tetrahedron Letters 13:3839-3842
19. Krygowski TM (1993) J Inf Comput Sci 33:70-78
20. Zborowski KK, Alkorta I, Elguero J, Proniewicz LM (2013) Struct Chem 24:543-548
21. Zborowski KK, Alkorta I, Elguero J, Proniewicz LM (2012) Struct Chem 23:595-600
22. Madura ID, Krygowski TM, Cyranski MK (1998) Tetrahedron 54:14913-14918
23. Krygowski TM (1993) J Chem Inf Comput Sci 33:70-78
24. Zborowski KK, Proniewicz LM (2009) Polish J Chem 83:477-484
25. Krygowski TM, Cyranski MK (1996) Tetrahedron 52:1713-1722
26. Krygowski TM, Cyrański M, Ciesielski A, Świrska B, Leszczyński P (1996) J Chem Inf Comput Sci 36:1135-1141
27. Bunn CW (1961) Chemical Crystallography. Clarendon Press, Oxford

28. Clar E (1964) Polycyclic Hydrocarbons. Vol. 1 and 2. Academic Press, London and New York; Springer Verlag, Berlin and Goettingen

29. Clar E (1972) The Aromatic Sextet. J. Wiley, Chichester

30. Krygowski TM, Ciesielski A, Bird CW, Kotschy A (1995) J Chem Inf Comput Sci 35:203-210

31. Zborowski KK, Alkorta I, Elguero J, Proniewicz LM (2012) Struct Chem 23:595-600

32. Cyranski MK, Krygowski TM, Katritzky AR, Schleyer PvR (2002) J Org Chem 67:1333-1338

33. Zborowski KK, Proniewicz LM (2009) Polish J Chem 83:477-484

34. Alonso M, Herradon B (2010) Phys Chem Chem Phys 12:1305-1317

35. Krygowski TM, Cyrański M (1996) Tetrahedron 52:10255-10264

36. Cyranski MK (1998) Analysis of the Aromatic Character of π -Electron Systems by Separating Geometric and Energetic Contributions. Ph.D. Thesis (in Polish), University of Warsaw, Warsaw

37. Bruice PY (2007) Organic chemistry. Pearson Prentice Hall, Upper Saddle River, p 580

38. Abraham RJ, Canton M, Reid M, Griffiths L(2000) J Chem Soc Perkin Trans2 803-812

39. Schleyer PvR, Maerker C, Dransfeld H, Jiao H, van Eikemma Hommes NJR (1996) J Am Chem Soc 118:6317-6318

40. Chen Z, Wannere CS, Corminboeuf C, Puchta R, Schleyer PvR (2005) Chem Rev 105:3842-3888

41. Mills NS, Llagostera KB (2007) J Org Chem 72:9163-9169

42. Flygare WH (1974) Chem Rev 74: 653-687

43. Dauben Jr HJ, Wilson JD, Laity JL (1968) J Am Chem Soc 90:811-813

44. Krygowski TM, Zachara JE, Szatylowicz H (2005) J Phys Org Chem 18:110-114

45. Krygowski TM, Szatylowicz H, Zachara JE (2004) J Chem Inf Comput Sci 44:2077-2082

46. Krygowski TM, Zachara JE, Szatylowicz H (2005) J Chem Inf Model 45:652-656

47. Hammett LP (1940) Physical Organic Chemistry. McGraw-Hill, New York, 1st Ed

48. Krygowski TM, Stepien BT (2005) Chem Rev 105:3482-3512

49. Ozimiński WP, Krygowski TM, Fowler PW, Sonchi A (2010) Org Lett 12:4880-4883

50. Streitwieser A Jr (1961) Molecular Orbital Theory for Organic Chemists. J. Wiley, New York, p. 237f

51. Skanke A (1971) In: Bergmann ED, Pullman B (eds) Aromaticity, Pseudoaromaticity, Antiaromaticity, Proceedings of an International Symposium held in Jerusalem 1970. Israel Academy of Science and Humanities, Jerusalem
52. Ciesielski A, Cyrański MK, Krygowski TM, Fowler PW, Lillington M (2006) J Org Chem 71:6840-6845
53. Fowler PW, Steiner E (1997) J Phys Chem A 101:1409-1413
54. Katritzky AR, Barczyński B, Musumurra G, Pisano D, Szafran M (1989) J Am Chem Soc 111:7-15
55. Jug K, Koester A (1991) J Phys Org Chem (1991) 4: 163-169
56. Katritzky AR, Karelson M, Sild S, Krygowski TM, Jug K (1998) J Org Chem 63:5228-5231
57. Bader RFW (1992) Atom in Molecules. A Quantum Theory. Oxford University Press, Oxford
58. Bader RFW (1991) Chem Rev 91: 893-928
59. Popelier P (2000) Atoms in Molecules, An Introduction. Printice Hall
60. Howard ST, Krygowski TM (1997) Can J Chem 75:1174-1181
61. Palusiak M, Krygowski TM (2007) Chemistry Eur J 13:7996-8006
62. Dominikowska J, Palusiak M (2012) Struct Chem 23:1173-1183
63. Poater J, Fradera X, Duran M, Sola M (2003) Chemistry Eur J 9:400-406
64. Matito E, Duran M, Sola M (2005) J Chem Phys 122:014109
65. Matta CF, Hernandez-Trujillo J (2005) J Phys Chem A 109:10798-10798
66. Bultinck P. Faraday Discuss. 2007, 135, 345-365
